# Allometry correction in semi-quantitative analysis of dopamine transporter SPECT

**DOI:** 10.1186/s13550-025-01353-0

**Published:** 2025-12-12

**Authors:** Thomas Buddenkotte, Ivayla Apostolova, Susanne Klutmann, Ralph Buchert

**Affiliations:** Department of Diagnostic and Interventional Radiology and Nuclear Medicine, University Medical Center Hamburg-Eppendorf, Martinistr. 52, 20246 Hamburg, Germany

**Keywords:** SPECT, Dopamine transporter, [^123^I]FP-CIT, Ioflupane, Specific binding ratio, Spatial normalization, Allometry, Allometric scaling, Putamen, Semi-quantitative analysis

## Abstract

**Background:**

Striatal specific binding ratios (SBR) are widely used to support the interpretation of dopamine transporter SPECT scans. Automatic SBR computation often involves using affine transformations to map the individual SPECT images to an anatomical reference space for ROI analysis using predefined standard masks. This does not account for differences in volumetric scaling between brain structures since, by definition, affine transformations preserve volume ratios. However, striatal volume has been reported to scale proportional to (intracranial volume)^0.4^, indicating particularly pronounced “negative” allometric scaling. This study aimed to investigate the impact of disregarding allometric scaling on putamen SBR, and to propose an easy-to-implement method to avoid this issue. 656 [^123^I]FP-CIT SPECT (67.2 ± 11.4y, 44.2% females, 52.1/47.9% with reduced/normal striatal signal according to visual interpretation by an expert reader) were spatially normalized by affine transformation to the anatomical reference space of the Montreal Neurological Institute. Unilateral putamen SBR were estimated using hottest voxels analysis in large putamen masks predefined in the reference space. For conventional hottest voxels analysis, the number of hottest voxels was fixed at 1,250 (equivalent to 10 ml, the mean putamen volume in healthy adults). For allometry correction, the number of hottest voxels was determined separately for each subject as 1,250*DET^(1−0.4)^, where DET is the Jacobian determinant of the affine transformation. To characterize the impact of the allometry correction on diagnostic performance, a data-driven Gaussian mixture model was employed.

**Results:**

Linear regression in the visually normal SPECT revealed a positive correlation between uncorrected putamen SBR and DET (Pearson’s *R* = 0.509, 95%-CI 0.422–0.587). The correlation was significantly (*p* < 0.00005) weaker when allometry-corrected ROI analysis was used (*R* = 0.285, 95%-CI 0.180–0.383). The effect size of the distance between reduced and normal putamen SBR as determined by the Gaussian mixture model was significantly larger with than without allometry correction (3.979 versus 3.335, one-sided *p* < 0.0001). When using the visual expert reading as diagnostic reference standard, the overall accuracy of the dichotomized SBR was significantly improved by allometry correction (from 94.4% to 96.2%, one-sided *p* = 0.026).

**Conclusion:**

The diagnostic performance of semi-quantitative SBR analyses involving affine transformations to an anatomical reference space can be enhanced through the application of the proposed allometry correction.

## Background

SPECT imaging using the dopamine transporter (DAT) ligand [^123^I]FP-CIT is widely used to aid the etiological diagnosis of uncertain parkinsonian syndromes and to distinguish dementia with Lewy bodies from Alzheimer’s disease [1–5].

The visual interpretation of [^123^I]FP-CIT SPECT images can be complemented by computing specific binding ratios (SBRs) for the semi-quantitative characterisation of [^123^I]FP-CIT binding to DAT in the striatum and striatal subregions [6–11]. This requires the anatomical delineation of the striatum and its subregions in the SPECT images. Automatic methods are generally favoured over manual delineation to eliminate intra- and inter-observer variability [12, 13]. Automatic methods often involve spatial normalisation of the patient’s SPECT image to an anatomical reference space, allowing the application of standard region-of-interest (ROI) masks of the striatum and its subregions that are predefined in the reference space. In DAT-SPECT, spatial normalisation most often is achieved by affine 12-parameter transformations comprising translation, rotation, scaling (in size) and shear [14–24]. High-dimensional elastic transformations that allow also high-frequency regional deformations are rarely used [25–27]. The rationale for this is (i) to account for the limited anatomical information in [^123^I]FP-CIT SPECT and (ii) to avoid artificial deformation of striata with Parkinson-like reduction of [^123^I]FP-CIT uptake (e.g., to better match normal striatal signal), which could affect the diagnostic performance of the SBR [26].

Affine transformations do not preserve absolute volumes of brain structures, but they do preserve *relative* volumes, that is, the volumes of all structures change by the same factor: the Jacobian determinant (DET) of the affine transformation. Therefore, using affine transformations for spatial normalization of [^123^I]FP-CIT SPECT images assumes that all relevant brain structures scale equally in volume.

**Fig. 1:**
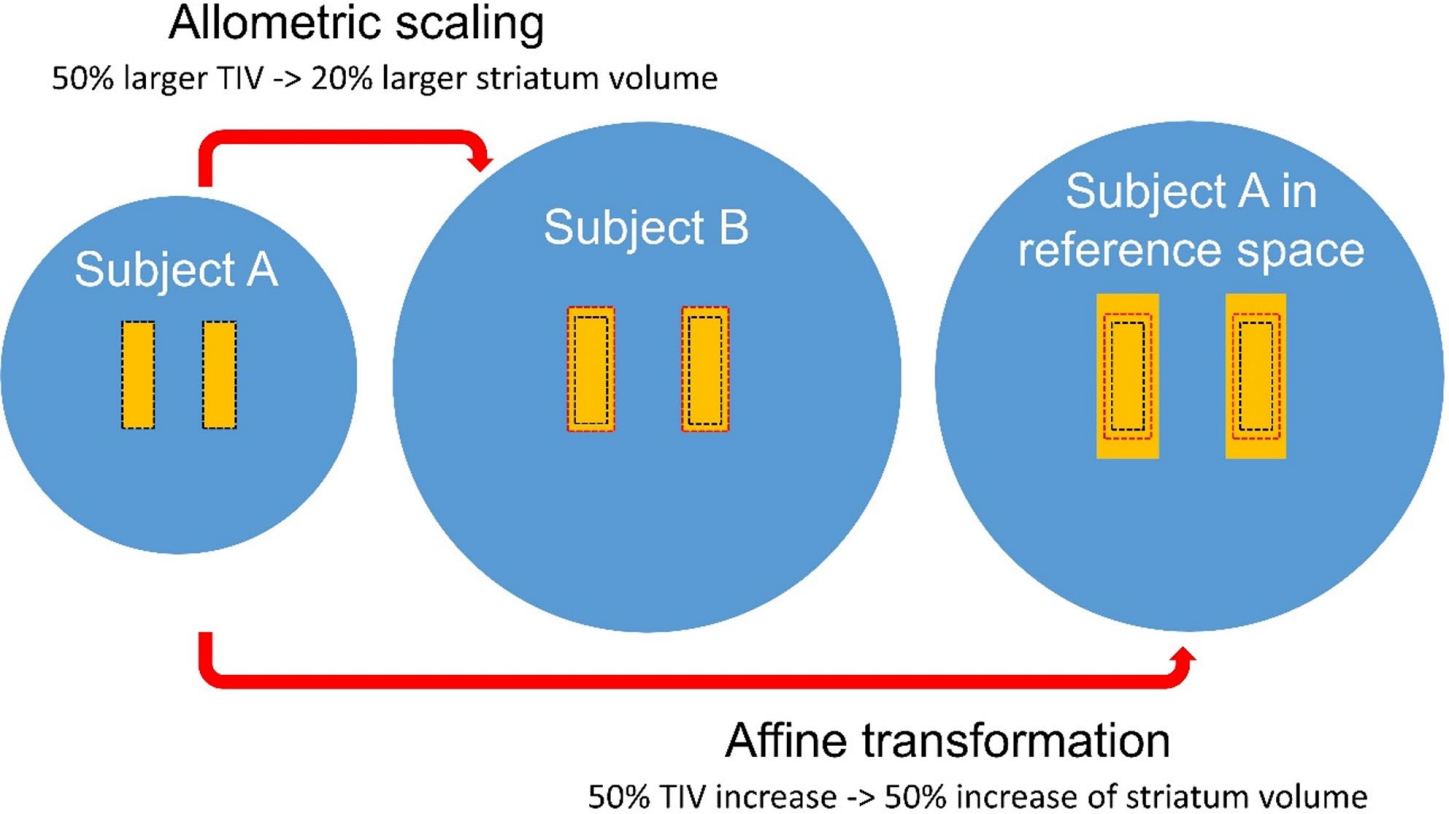
Allometric scaling of the striatum. The volume of the striatum shows strongly “negative” allometric scaling proportional to (intracranial volume)0.4. For example, if the total intracranial volume (TIV, blue) of subject B (middle) is 50% larger than that of subject A (left), the striatal volume (orange) is on average only about 20% larger in subject B. Affine transformations preserve volume ratios and therefore result in 50% increase of the striatal volume when the TIV is increased by 50% (right). The black dashed rectangle represents the striata of subject A, the red dashed rectangle represents the striata of subject B. The affine transformation of subject A (to the space of subject B) results in an about 25% too large striatum volume on average (orange area outside the red dashed rectangle in subject A after affine transformation, right). Thus affine transformations in general result in striata of unnatural size. The allometry correction proposed in this study accounts for this by adjusting the number of hottest voxels included for SBR estimation (“more hottest voxels when the striatum is too large after affine transformation, less hottest voxels when the striatum is too small”)

However, there is growing evidence that brain structures exhibit allometric scaling in relation to brain size, that is, the relationship between the volume of a specific brain structure and total intracranial volume (TIV) may be non-proportional and non-linear [28, 29]. De Jong and co-workers, using fully automated volumetry based on multispectral MRI to investigate allometric scaling in a large sample of community-dwelling older adults, reported the striatum volume to scale proportional to $\:{TIV}^{\alpha\:}$ with an age- and sex-corrected allometry coefficient α of approximately 0.4 (95% confidence interval: 0.37–0.45) [28]. This indicates strongly “negative” allometric scaling of the striatum. For example, if the TIV of patient B is 50% larger than that of patient A, the striatal volume of patient B is only about 20% larger on average. Conventional semi-quantitative analyses in dopamine transporter SPECT based on affine transformations disregard this effect entirely: when the affine transformation of an individual [^123^I]FP-CIT SPECT image from patient to reference space results in a 50% increase of the TIV (and consequently of the striatal volume, too), the volume of the striata is overestimated (relative to the TIV) in the reference space by about 25% on average (Fig. 1).

Against this background, the current study aimed (i) to investigate the impact of disregarding allometric scaling on the putamen [^123^I]FP-CIT SBR, and (ii) to propose an easy-to-implement method to avoid this issue. The SBR of the putamen was considered rather than that of the whole striatum, because Parkinson-like reduction of [^123^I]FP-CIT uptake is more pronounced in the putamen than in the caudate nucleus [3]. Consequently, the SBR of the putamen generally provides better diagnostic power than that of the whole striatum [21].

## Methods

### Patients

This retrospective study screened 671 consecutive patients who were referred to [^123^I]FP-CIT SPECT due to a clinically uncertain parkinsonian syndrome or suspected dementia with Lewy bodies. Fifteen of these patients were excluded because of relevant mass lesions in the striata. There were no further eligibility criteria to ensure that the included patient sample was representative of clinical routine. This resulted in the inclusion of 656 patients (44.2% female, 67.2 ± 11.4y, range 26.4–91.6y). From these, 342 (52.1%) showed Parkinson-like reduction of the striatal signal according to the retrospective visual interpretation by an experienced reader (20 years, ≥ 5,000 cases). The remaining 314 (47.9%) [^123^I]FP-CIT SPECT were categorized as normal by the experienced reader. The experienced reader’s visual interpretation served as the reference standard against which the diagnostic accuracy of putamen SBR was compared, both with and without correction for allometric effects.

Waiver of informed consent for the retrospective analysis of the anonymized data was obtained from the ethics review board of the general medical council of the state of Hamburg, Germany. All procedures were in accordance with the ethical standards of the ethics review board and with the 1964 Helsinki declaration and its later amendments.

Most of the patients were previously included in a study on the scan duration in [^123^I]FP-CIT SPECT with multiple-pinhole (MPH) collimators [14].

### SPECT

SPECT acquisition in list mode started 3.4 ± 0.4 h after intravenous injection of 181 ± 12 MBq [^123^I]FP-CIT. A total of 90 projection views (30 per head, 120° scan arc) with angular steps of 4° were acquired in list mode using a triple-head general-purpose camera (AnyScan Trio S, Mediso Medical Imaging Systems, Budapest, Hungary) equipped with second-generation general-purpose brain MPH collimators [17]. The energy window was set to 143–175 keV. The distance between the center-of-rotation axis and the pinhole focal plane was fixed at 150 mm. Helical acquisition mode with 40 mm total table displacement was used to avoid axial undersampling [30, 31].

Projection data corresponding to 12 min total net scan duration were sorted into 256 × 256 matrices with 2.13 × 2.13 mm^2^ pixel size [14].

Transaxial images consisting of cubic voxels of 3.6 mm edge length were reconstructed using the Tera-Tomo Monte Carlo photon simulation engine with 300 effective iterations (3 subsets, 100 iterations) of the one-step-late maximum-a-posteriori expectation-maximization algorithm implemented in the camera software [32–34].

Correction for photon attenuation and scatter was not performed, because it does not affect the ability of [^123^I]FP-CIT SPECT to distinguish between normal and Parkinson-like reduction of the striatal signal [15].

### Image preprocessing

Individual [^123^I]FP-CIT SPECT images were spatially normalized to the anatomical space of the Montreal Neurological Institute (MNI) by affine 12 parameters transformation using the Normalize tool of the Statistical Parametric Mapping software package (version SPM12) and a set of custom [^123^I]FP-CIT SPECT templates representative of normal and different levels of Parkinson-typical reduction of striatal uptake as target [16]. The voxel size of the spatially normalized images was 2 × 2 × 2 mm^3^.

Intensity normalization was achieved by voxelwise scaling to the individual 75th percentile of the voxel intensity in a reference region comprising the whole brain parenchyma without striata, thalamus, brainstem and cerebellum [35]. The scaled images are semi-quantitative images representing the distribution volume ratio (DVR).

### Semi-quantitative analysis

The (uncorrected) SBR of [^123^I]FP-CIT in left and right putamen were obtained by hottest voxels analysis of the spatially normalized DVR image using unilateral, three-dimensional ROI masks predefined in MNI space [20]. Each unilateral ROI mask had a volume of 56.9 ml and, therefore, was much bigger than the anatomical putamen. The rationale for this was to guarantee that all counts from the putamen were included in the ROI mask, despite mislocalization of striatal counts due to limited spatial resolution and residual anatomical intersubject variability after spatial normalization. The number of hottest voxels within a unilateral putamen mask was fixed to a total volume of 10 ml (= typical putamen volume in healthy adults [36]), corresponding to 1,250 cubic voxels of 2 mm edge length in the spatially normalized DVR image. Thus, each unilateral putamen SBR was estimated as the mean DVR across the 1,250 hottest voxels in the corresponding unilateral putamen mask minus 1. The minimum of the putamen SBR of both hemispheres was used in all analyses because it provides better discriminative power than the mean [16].

### Allometry correction

The underlying concept of the proposed allometry correction is to account for artificially over- or undersized striata (relative to the TIV) after affine transformation by increasing or decreasing the number of hottest voxels to be averaged for the SBR estimation from the default of 1,250. More precisely, the number of hottest voxels to be used was computed according to.

1 $$\begin{aligned} \text{number of hottest voxels} & = 1,250 \:{}^* \: \mathrm{DET}^{(1-\alpha) } \\ & = 1,250\:{}^*\: \mathrm{DET}^{0.6} \end{aligned}$$

where DET is the Jacobian determinant of the affine transformation and ɑ is the striatal allometry coefficient, reported to be approximately 0.4 [28]. Thus, the number of hottest voxels to be used for the SBR estimation varied between patients. For example, when the TIV (and striatum volume) was doubled by the affine transformation (DET = 2), approximately 1,900 voxels were used rather than 1,250.

### DET-adjustment of the SBR

In the 314 normal [^123^I]FP-CIT SPECT according to the visual expert categorization, linear regression of the (uncorrected) putamen SBR was performed with DET as predictor:

2 $$\mathrm{SBR} = \mu \: {}^* \: \mathrm{DET} + \mathrm{const}$$

Then, the SBR was adjusted for DET as.

3 $$\mathrm{SBRadj} = \mathrm{SBR} - \mu \: {}^* \: (\mathrm{DET} - \text {meanDET})$$

where meanDET is the mean value of DET across the 314 normal [^123^I]FP-CIT SPECT.

In the current study, DET-adjustment of the SBR was used as benchmark for the proposed allometry correction of the ROI analysis. We do not propose using DET-adjustment for allometry correction, because achieving optimal performance likely requires a large, camera-specific dataset of normal [¹²³I]FP-CIT SPECT images for camera-specific µ estimation. While such a dataset was available for the current study, it may not be readily accessible at all institutions.

### Statistical analysis

The dependence of the putamen SBR on DET was tested by linear regression in the [^123^I]FPCIT SPECT categorized as normal by the experienced reader (*n* = 314). This was performed separately for uncorrected, allometry-corrected and DET-adjusted SBR. For statistical comparison of the uncorrected SBR and the allometry-corrected SBR regarding their correlation with DET, the online version of the „comparing correlations (cocor)“ R programming language package [37] was used (http://comparingcorrelations.org/, setting: overlapping correlations in the same group).

The impact of the allometry correction on the power of the putamen SBR to discriminate between reduced and normal [^123^I]FPCIT SPECT was tested using a data-driven Gaussian mixture model approach as described previously [14, 15]. In brief, the distribution of the putamen SBR was characterized by a histogram with bin width of 0.1 and fitted by the sum of two Gaussians:

4 $$\begin{aligned} \:histogram\left(SBR\right) & ={A}_{1}exp\left(-\frac{{\left(SBR-{M}_{1}\right)}^{2}}{{2SD}_{1}^{2}}\right)\\ & \quad +{A}_{2}exp\left(-\frac{{\left(SBR-{M}_{2}\right)}^{2}}{{2SD}_{2}^{2}}\right) \end{aligned}$$

where *A*_*1*_, *A*_*2*_ are the amplitudes, *M*_*1*_, *M*_*2*_ are the mean values and *SD*_*1*_, *SD*_*2*_ are the standard deviations of the Gaussian functions. The MATLAB routine ‘fminsearch’ with default parameter settings was used for this purpose.

The discriminative power of the putamen SBR was estimated by the effect size *d* of the distance between the two Gaussians computed as the differences between their mean values scaled to the pooled standard deviation.

5 $$\:d=\left({M}_{2}-{M}_{1}\right)/\sqrt{\frac{{SD}_{1}^{2}+{SD}_{2}^{2}}{2}}$$

The cutoff *c* for dichotomization of the SBR to discriminate between reduced and normal values was selected halfway between *M*_*1*_ and *M*_*2*_ in units of standard deviations, that is.

6 $$\:c=\left({{SD}_{2}M}_{1}+{{SD}_{1}M}_{2}\right)/\left({SD}_{1}+{SD}_{2}\right)$$

The SBR was dichotomized using *c* as cutoff: an SBR was considered normal if SBR ≥ *c*, it was considered reduced if SBR < *c*. Then, overall accuracy, sensitivity and specificity of the dichotomized SBR was computed relative to the visual expert categorization as reference standard.

The Gaussian mixture model analysis was performed separately for uncorrected, allometry-corrected and DET-adjusted SBR. Custom MATLAB scripts were used (MATLAB version 2017b).

In order to estimate 95% confidence intervals (95%-CI) for the parameters of the Gaussian mixture model and the metrics derived from it, bootstrapping with 10,000-fold resampling was used. More precisely, 10,000 random samples, each comprising 656 cases, were drawn from the 656 included patients with replacement. The same random samples were used for uncorrected, allometry-corrected and DET-adjusted SBR. For each parameter, the 95%-CI was estimated as the interval from the 2.5th to the 97.5th percentile across the 10,000 random samples. Statistical significance of differences between without and with allometry correction was tested by counting the number of ‘allometry-corrected < uncorrected’ cases across the 10,000 random samples.

## Results

In the 314 normal [^123^I]FP-CIT SPECT according to the visual expert categorization, the DET of the affine transformation ranged between 0.916 and 1.986 (mean ± standard deviation = 1.408 ± 0.205).

Pearson’s correlation coefficient between uncorrected and allometry-corrected SBR in the normal SPECT scans (*n* = 314) was *R* = 0.967 (95%-CI 0.960–0.974).

**Fig. 2:**
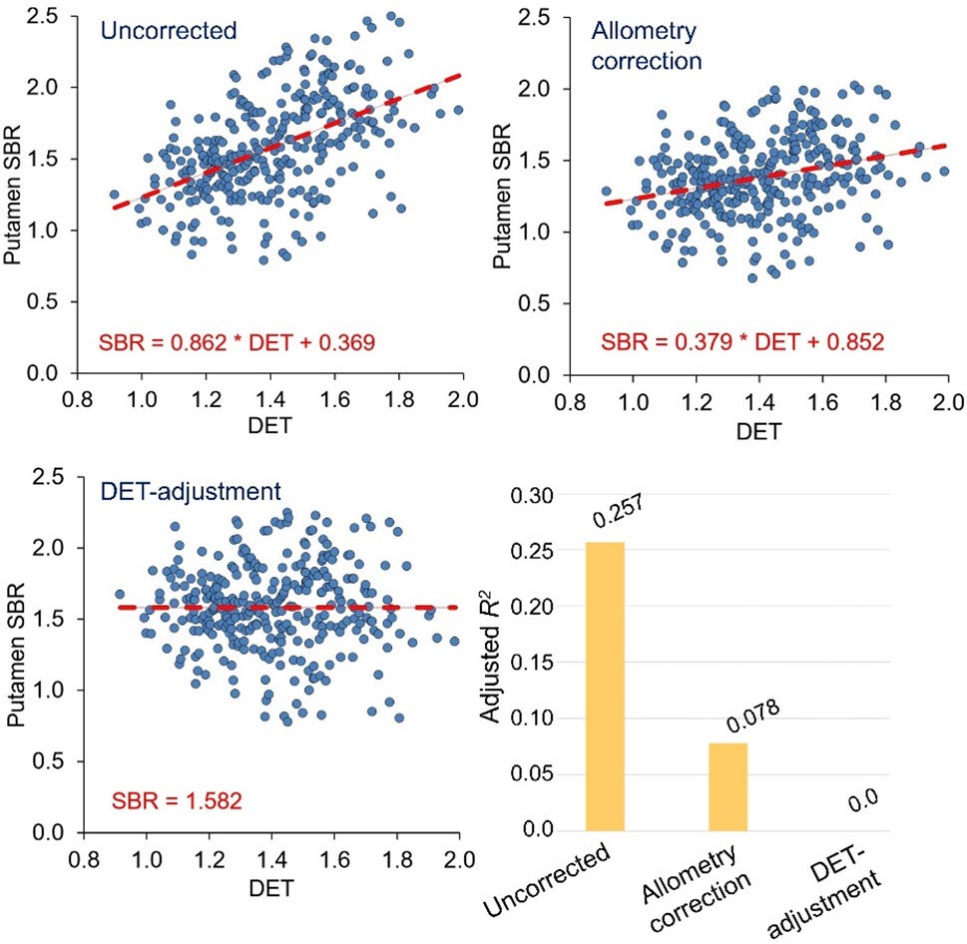
Scatter plot of putamen SBR versus DET. Scatter plot of the putamen SBR (minimum of both hemispheres) in the normal cases (according the visual expert read) without allometry correction (top left), with allometry correction (top right) and with DET-adjustment of the SBR (bottom left) versus the amount of stretching (DET) by the affine transformation. The dashed lines indicate linear regression. The coefficient of determination (R^2^) of the putamen SBR by the DET is shown on the bottom right

The results of the linear regression of the SBR in the normal [^123^I]FPCIT SPECT with DET as predictor variable are summarized in Fig. 2. The uncorrected SBR was positively correlated with the DET (*R* = 0.509, 95%-CI 0.422–0.587; adjusted *R*^*2*^ = 0.257; slope = 0.862; standardized regression coefficient ß = 0.509; *p* < 0.001). The correlation was significantly (two-sided *p* < 0.00005) weaker when the allometry-corrected ROI analysis was used to estimate the SBR (*R* = 0.285, 95%-CI 0.180–0.383; adjusted *R*^*2*^ = 0.078; slope = 0.379; ß = 0.285; *p* < 0.001). The 95%-CI for the difference between the two correlation coefficients was 0.197–0.260 (Zou’s method). DET-adjustment of the SBR according to Eq. (3) with µ = 0.862 and meanDET = 1.408 eliminated the correlation (by design).

**Fig. 3:**
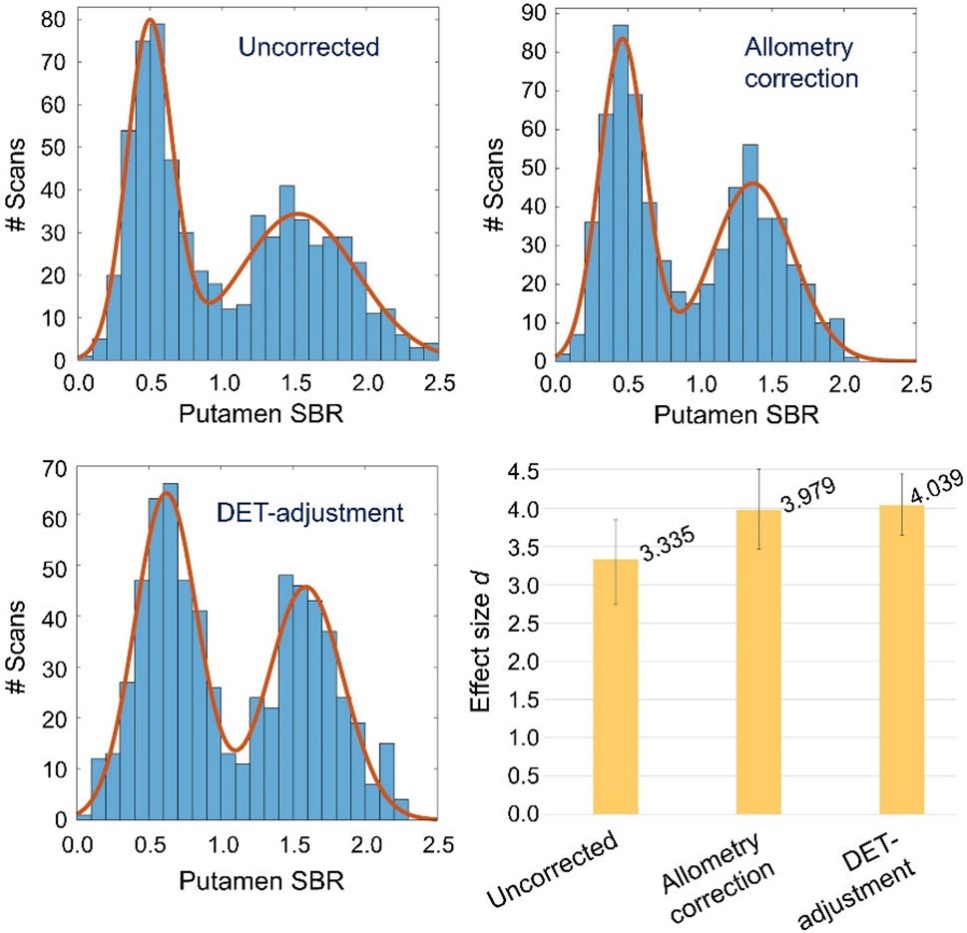
Gaussian mixture model analysis. Histograms of the putamen SBR without allometry correction (top left), with allometry correction (top right) and with DET-adjustment of the SBR (bottom left). The continuous lines indicate the fit by the Gaussian mixture model. The effect size *d* of the distance between the two Gaussian functions (according to Eq. (5)) is shown on the bottom right (mean value across the 10,000 random samples for bootstrapping). The error bars indicate the 95% confidence interval estimated by bootstrapping (Fig. 4)

**Fig. 4:**
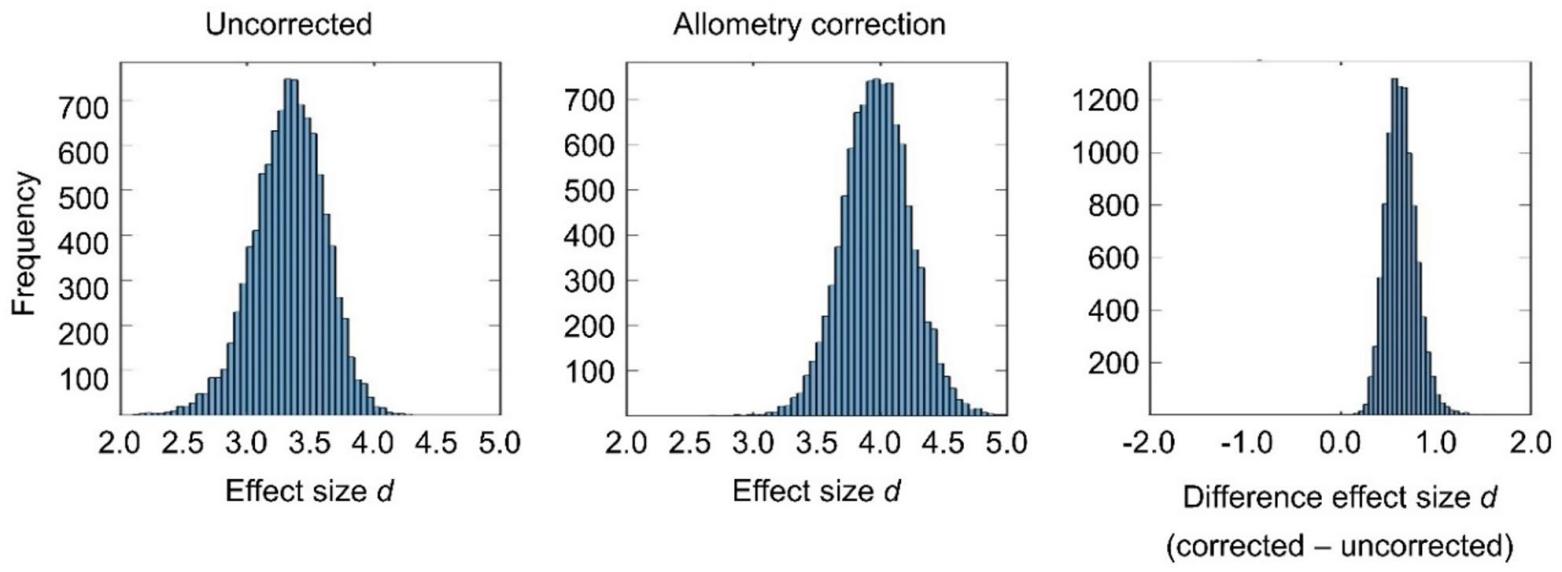
Bootstrapping of the data-driven Gaussian mixture model analysis. Histograms of the effect size d of the distance between ‘reduced’ and ‘normal’ SBR without (left) and with (middle) allometry correction across the 10,000 bootstrapping random samples of the data-driven Gaussian mixture model approach. The histogram of the difference in effect size between with and without allometry correction across the 10,000 random samples is shown on the right

**Fig. 5:**
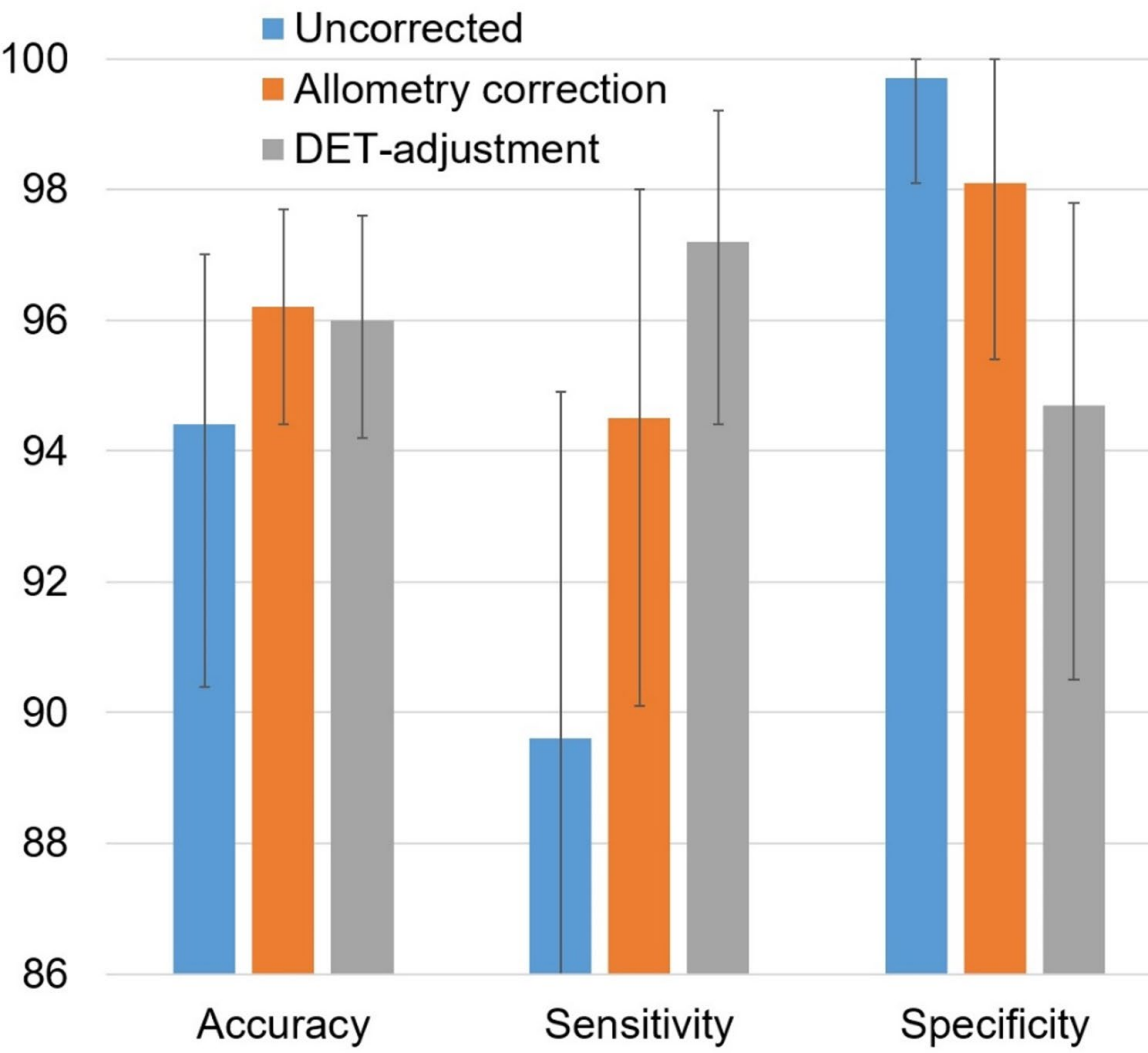
Overall accuracy, sensitivity and specificity (in %) of the putamen SBR (minimum of both hemispheres) without allometry correction (blue), with allometry correction (orange) and with DET-adjustment of the SBR (gray). Dichotomization of the SBR values was performed using the cutoff c from the Gaussian mixture model fit of the corresponding SBR histogram. The visual expert read was used as reference standard. Shown are the mean values across the 10,000 random samples for bootstrapping. The error bars represent 95%-confidence intervals estimated by bootstrapping.

The results of the Gaussian mixture model analysis for the data-driven characterization of the impact of allometry correction on the power to discriminate between normal and reduced putamen SBR are summarized in Table 1; Figs. 3, 4 and 5. The effect size *d* of the distance between ‘reduced’ and ‘normal’ SBR was smaller with than without allometry correction in none of the 10,000 bootstrapping random samples, indicating its improvement by allometry correction, from *d* = 3.335 on average to *d* = 3.979 on average, to be statistically significant at the one-sided *p* < 0.0001 level. DET-adjustment of the SBR resulted in a further increase of the effect size to *d* = 4.039 on average (Table 1). The improvement in effect size by allometry correction was mainly driven by narrowing the distribution of normal SBR (reduction of CoV2 from 26.8% to 20.5%, Table 1; Fig. 3). Allometry correction also resulted in a widening of the distribution of reduced SBR (increase of CoV1 from 31.4% to 34.3%; Table 1), but this effect was less pronounced and less obvious visually (Fig. 3) than the narrowing of the distribution of normal SBR.

**Table 1:** Gaussian mixture model analysis of the putamen SBR

	Uncorrected	Allometry-corrected	DET-adjusted
Reduced: *M1*	0.498 (0.474–0.523)	0.463 (0.440–0.486)	0.621 (0.590–0.652)
Reduced: *SD1*	0.157 (0.131–0.182)	0.159 (0.136–0.182)	0.217 (0.188–0.248)
Reduced: *CoV1* (%)	31.4 (26.7–36.0)	34.3 (29.6–39.0)	35.0 (30.3–39.8)
Normal: *M2*	1.525 (1.455–1.587)	1.367 (1.326–1.405)	1.588 (1.548–1.627)
Normal: *SD2*	0.409 (0.345–0.488)	0.280 (0.232–0.332)	0.260 (0.214–0.310)
Normal: *CoV2* (%)	26.8 (22.3–33.1)	20.5 (16.9–24.6)	16.4 (13.5–19.6)
Effect size *d*	3.335 (2.742–3.853)	3.979 (3.462–4.508)	4.039 (3.649–4.442)
Cutoff *c*	0.784 (0.703–0.859)	0.791 (0.720–0.859)	1.062 (0.984–1.135)
Accuracy (%)	94.4 (90.4–97.0)	96.2 (94.4–97.7)	96.0 (94.2–97.6)
Sensitivity (%)	89.6 (81.7–94.9)	94.5 (90.1–98.0)	97.2 (94.4–99.2)
Specificity (%)	99.7 (98.1–100)	98.1 (95.4–100)	94.7 (90.5–97.8)

Mean values M1, M2 and standard deviations SD1, SD2 from the fit of the histogram of the putamen SBR (minimum of both hemispheres, n = 656) by the sum of two Gaussians according to Eq. (4), effect size d of the distance between the two Gaussian functions (the first representing reduced SBR, the second representing normal SBR) according to Eq. (5), and cutoff c for the dichotomization of SBR as normal or reduced according to Eq. (6). The coefficients of variance (CoV) were computed as 100*SD/M to compare the relative width between both Gaussians. The analysis was performed separately without and with allometry correction and with DET-adjustment of the putamen SBR. The specified values represent the averages obtained from 10,000 random samples used for bootstrapping. The ranges in brackets represent the estimated 95% confidence intervals, calculated using the 2.5th and 97.5th percentiles across the 10,000 random samples

Overall accuracy of the dichotomized SBR (based on the cutoff *c* from the Gaussian mixture model) relative to the visual expert categorization was smaller with than without allometry correction in 259 of the 10,000 bootstrapping random samples, indicating its improvement by allometry correction, from 94.4% on average to 96.2% on average, to be statistically significant at the one-sided *p* = 0.026 level. The dichotomized version of the DET-adjusted SBR provided overall accuracy of 96.0%, that is, very similar to allometry-corrected SBR.

## Discussion

The first key finding of this study was that semi-quantitative analysis of [^123^I]FP-CIT SPECT based on spatial normalization with affine transformations results in a *positive* correlation of the (uncorrected) putamen SBR with the Jacobian determinant (DET) of the affine transformation. The current study observed this in a large, homogenous dataset of 656 high-resolution [^123^I]FP-CIT SPECT images, all of which were acquired with the same triple-head camera equipped with second-generation, general-purpose brain MPH collimators [17]. It confirms the findings of a previous study using an even larger, albeit less homogeneous, dataset of 1,702 standard-quality [¹²³I]FP-CIT SPECT images acquired using four different cameras equipped with either low-energy-high-resolution parallel-hole or fan-beam collimators [16]. The authors of this previous study hypothesized that the positive correlation between the putamen SBR and the DET could be driven by between-cameras variability in spatial resolution, at least to some extent [16]. The current study demonstrates that between-subjects variability of spatial resolution in the reconstructed SPECT images is most likely not the main driver of the positive correlation between the uncorrected putamen SBR and DET.

In the 314 [¹²³I]FP-CIT SPECT that were categorized as normal by the experienced reader, the weak to moderate correlation with the DET explained approximately 25% of the between-subjects variability of the (uncorrected) putamen SBR (adjusted *R*^*2*^ = 0.257). This additional, technical between-subjects variability could reduce the discriminative power of the putamen SBR to detect nigrostriatal degeneration.

The *positive* correlation between putamen SBR and DET corresponds to a *negative* correlation between putamen SBR and patients’ head size, because higher DET indicate smaller brains that require more stretching to transform them to the anatomical reference space. This *negative* correlation cannot be explained by partial volume/recovery effects due to limited spatial SPECT resolution. Partial volume/recovery effects are expected to result in a *positive* correlation between putamen SBR and head size (“the smaller the head, the stronger the underestimation of the SBR”) [16].

The second key finding of this study was that applying the proposed allometry correction significantly weakened the correlation between normal SBR and DET: the amount of normal between-subjects variability that was explained by the DET was reduced by the allometry correction from approximately 25% without correction to approximately 8% with correction. The resulting reduction in the between-subjects variability of normal putamen SBR was clearly noticeable through a narrower distribution, both visually (Fig. 3) and in the Gaussian mixture model analysis (Table 1). The impact of the allometry correction on the (relative) width of the distribution of *reduced* putamen SBR was less striking. This is probably because the distribution of reduced putamen SBR is wider in general due to the variability in the severity of nigrostriatal degeneration between patients.

Consistent with the reduction of between-subjects variability of normal putamen SBR by the allometry-correction, the effect size *d* of the distance between reduced and normal SBR in the Gaussian mixture model increased by approximately 20% (from 3.335 to 3.979; Table 1). This suggests that the allometry-corrected putamen SBR can improve diagnostic power compared to the uncorrected SBR. This was confirmed by comparing the dichotomized putamen SBR with the expert reading as reference standard: overall accuracy of SBR-based categorization increased significantly from 94.4% without correction to 96.2% with allometry correction (Fig. 5).

Although the proposed allometry correction weakened the correlation between putamen SBR and DET considerably, it did not eliminate it entirely. This is not surprising, given that the method of correcting for the failure of affine transformations to account for allometric scaling, by varying the number of voxels used for SBR estimation, is not expected to be exact.

Furthermore, the age- and sex-corrected allometry coefficient (α = 0.4) reported by de Jong et al. for the *whole* striatum was used for the allometry-corrected ROI analysis of the putamen (Eq. (1)). This could have resulted in undercorrection, given that there is some evidence suggesting that allometric scaling is slightly more pronounced in the putamen than in the caudate nucleus [29].

However, the DET-adjustment of the putamen SBR (to eliminate DET dependency entirely) only increased the effect size (*d*) by approximately 1.5% in the Gaussian mixture model analysis compared to the allometry-corrected ROI analysis (from 3.979 to 4.039, Table 1). The diagnostic performance (relative to the visual expert read) after dichotomization was also very similar: 96.0% overall accuracy with DET-adjustment versus 96.2% with allometry correction (Table 1; Fig. 5). These findings suggest that the limitations of the allometry correction only have a minor impact on the diagnostic performance of the putamen SBR, although sensitivity and specificity were slightly more balanced with DET-adjustment (97.2% and 94.7%) than with allometry correction (94.5% and 98.1%, Table 1; Fig. 5). Small imbalance in sensitivity versus specificity could be reduced without significant loss of overall accuracy by adjusting the cutoff for SBR dichotomization.

We hypothesise that the proposed allometry correction, as defined by Eq. (1), can be applied to other acquisition and reconstruction settings unchanged. Therefore, we recommend its use in clinical practice and research. In contrast, achieving optimal performance of DET-adjustment according to Eqs. (2) and (3) in general requires a sufficiently large, camera-specific dataset of normal [¹²³I]FP-CIT SPECT images for µ estimation. Such a dataset is not accessible at all institutions. However, DET-adjustment with fixed µ could outperform the proposed allometry-correction approach not only in mono-site/mono-camera settings but also in multi-site/multi-camera settings when using harmonized acquisition and reconstruction protocols. The added value regarding the binary categorization of [¹²³I]FP-CIT SPECT scans for detection (or exclusion) of nigrostriatal degeneration, compared to the proposed allometry correction, is likely to be rather small. Even in the mono-site/mon-camera setting of the current study, the overall accuracy of the dichotomized SBR was very similar with allometry correction and DET-adjustment (Table 1; Fig. 5).

The proposed allometry correction changes the number of voxels in the ROI analysis to estimate the mean [^123^I]FP-CIT retention in the putamen. This method can be easily implemented when large putamen masks and hottest voxels analysis are used. It is less straightforward when using anatomical ROI masks intended for the correct anatomical delineation of the putamen.

A secondary finding of this study was that the mean DET of the affine transformation across all normal [¹²³I]FP-CIT SPECT scans was approximately 1.4, indicating an average enlargement of brain volume by 40%. This is consistent with the MNI brain being significantly larger than the average brain [38].

The fact that the reference standard used to estimate the diagnostic performance of the dichotomised SBR (and the impact of the allometry correction on it) was based on the visual interpretation of the [¹²³I]FP-CIT SPECT images by a single reader is a limitation of the current study. However, the proportion of [¹²³I]FP-CIT SPECT scans with discrepant binary visual categorisation by two independent expert readers is expected to be small (typically ≤ 3% [15]). Furthermore, limitations of the reference standard for estimating the diagnostic performance of the dichotomised SBR do not affect the other metrics used in this study to test the proposed allometry correction: (i) the correlation between SBR and DET, and (ii) the separation between reduced and normal SBR in the data-driven Gaussian mixture model analysis. Thus, the lack of additional readers most likely did not have a significant impact on the study’s results and conclusions.

## Conclusions

Using affine transformations to enable the use of predefined standard masks for ROI analysis disregards the significant allometric scaling of the putamen in the semi-quantitative analysis of [¹²³I]FP-CIT SPECT. This not only results in a spurious positive correlation between the putamen SBR and the Jacobian determinant of the affine transformation, but also causes additional between-subjects variability of no interest in the putamen SBR that can affect its diagnostic power. The latter can be largely avoided through an easy-to-implement allometry correction that is based on adjusting the number of voxels used for SBR estimation for each patient to account for the incorrect size of the striatum after affine transformation.

## Data Availability

The results of the semi-quantitative analyses can be made available in anonymized form on reasonable request.

## Abbreviations

95%-CI: 95% confidence interval
ɑ: allometry coefficient
[^123^I]FP-CIT: N-ω-fluoropropyl-2β-carbomethoxy-3β-(4-I-123-iodophenyl)nortropane
DAT: Dopamine transporter
DVR: Distribution volume ratio
MNI: Montreal neurological institute
MPH: Multiple-pinhole
R: Pearson’s correlation coefficient
ROI: Region-of-interest
SBR: Specific binding ratio
SPECT: Single-photon emission computed tomography
SPM: Statistical parametric mapping
TIV: Total intracranial volume
